# DRaW: prediction of COVID-19 antivirals by deep learning—an objection on using matrix factorization

**DOI:** 10.1186/s12859-023-05181-8

**Published:** 2023-02-15

**Authors:** S. Morteza Hashemi, Arash Zabihian, Mohsen Hooshmand, Sajjad Gharaghani

**Affiliations:** 1 Department of Computer Science and Information Technology, Institute of Advanced Studies in Basic Sciences, Zanjan, Iran; 2 Laboratory of Bioinformatics and Drug Design, University of Tehran, Tehran, Iran; 3 Department of Bioinformatics, Kish International Campus, University of Tehran, Kish, Iran

**Keywords:** Drug repurposing, Matrix factorization, Deep learning, COVID-19

## Abstract

**Background:**

Due to the high resource consumption of introducing a new drug, drug repurposing plays an essential role in drug discovery. To do this, researchers examine the current drug-target interaction (DTI) to predict new interactions for the approved drugs. Matrix factorization methods have much attention and utilization in DTIs. However, they suffer from some drawbacks.

**Methods:**

We explain why matrix factorization is not the best for DTI prediction. Then, we propose a deep learning model (DRaW) to predict DTIs without having input data leakage. We compare our model with several matrix factorization methods and a deep model on three COVID-19 datasets. In addition, to ensure the validation of DRaW, we evaluate it on benchmark datasets. Furthermore, as an external validation, we conduct a docking study on the COVID-19 recommended drugs.

**Results:**

In all cases, the results confirm that DRaW outperforms matrix factorization and deep models. The docking results approve the top-ranked recommended drugs for COVID-19.

**Conclusions:**

In this paper, we show that it may not be the best choice to use matrix factorization in the DTI prediction. Matrix factorization methods suffer from some intrinsic issues, e.g., sparsity in the domain of bioinformatics applications and fixed-unchanged size of the matrix-related paradigm. Therefore, we propose an alternative method (DRaW) that uses feature vectors rather than matrix factorization and demonstrates better performance than other famous methods on three COVID-19 and four benchmark datasets.

## Introduction

Drug discovery is a highly sensitive, with public domain aspect to the research that needs a tremendous amount of time and cost [1, 2]. Thus, scientists and researchers take advantage of computational methods in drug discovery [3, 4]. Drug-repurposing is one of its main branches that finds new indications for approved drugs [5]. This point of view is constructive, especially in an urgent situation, e.g., the coronavirus disease (COVID-19) pandemic [6–8].

Computational drug-repurposing methods, applied to COVID-19, can be categorized into three groups: (i) network-based methods; (ii) structure-based methods; and (iii) machine learning (ML)-based methods [9]. The methods of the first group, network-based methods, identify proteins that are functionally related to COVID-19. Messina et al. [10] studied the interactome of human coronaviruses (HCoV) with their host cells using a network-based model simulation. They utilized curated protein-protein interactions and gene co-expression data to analyze all possible virus-host protein interactions. Sadegh et al. [11] used a network-based technique to investigate the SARS-CoV-2 virus-host-drug interactome in order to predict repurposable treatment candidates. To that purpose, they created the CoVex online platform, which incorporates drug-target interaction and PPIs data to help with the drug repurposing process.

The methods of the second group, structure-based techniques, investigate the possible interactions between therapeutic agents and macromolecular targets in order to discover new uses for existing drugs. Culletta et al. [12] looked for potential therapeutics against SARS-CoV-2 using a structure-based pharmacophore modeling technique. They investigated the SARS-CoV-2 proteome and identified high-quality protein models using homology modeling. Also, to discover pharmacophore features for each target, they conducted structure-based modeling. Then, the obtained results were employed in a series of virtual screenings against the DrugBank database. Following a docking study, they discovered a total of 34 hits for all of the investigated targets, and the potential drugs were chosen based on the best binding energy for each drug as determined by the molecular mechanics with generalized born and surface area solvation (MM/GBSA) calculation. Juárez-Saldívar and colleagues [13] performed a virtual screening of four databases (PDB, ChEMBL, BindingDB, and DrugBank) to identify potential SARS-CoV-2 main protease (Mpro) inhibitors. They investigated the binding affinity of chemical compounds and Mpro using the docking approach. The candidate compounds were then clustered based on structural differences in order to uncover structural features of potential SARS-CoV-2 inhibitors. In addition to the aforementioned investigations, more recent studies on structure-based drug repurposing have focused on the targetability of the spike protein as a potential candidate to inhibit the SARS-CoV-2-ACE2 receptor [14–16].

The last group is the ML drug repurposing approaches. Beck and colleagues [17] developed a deep learning model for predicting drug-protein binding affinity based on the molecular transformer-drug target interaction (MT-DTI). Using this model, they discovered that atazanavir, remdesivir, and efavirenz are effective inhibitors against SARS-CoV-2 3C-like proteinase. Tian et al. [18] suggested a unique drug repositioning approach (called VDA-KLMF). This suggested model incorporates information from known viral-drug associations, drug chemical structures, and virus sequences. Gaussian kernels of viruses and drugs are generated using known viral-drug associations. Then, by utilizing biological features and an identity matrix, the similarity kernels of viruses and drugs were generated. In the next step, the similarity and Gaussian kernels are diffused, and a logistic matrix factorization model with kernel diffusion was suggested to find possible anti-SARS-CoV-2 drugs. In another study, Zeng et al. [19] developed an integrative strategy that combines network-based and deep learning techniques, to predict drugs for COVID-19. They created an extensive knowledge graph with 15 million connections linking drugs, diseases, proteins or genes, pathways, and expressions from a significant collection of scientific literature. Their suggested model predicted 41 repurposable drugs. In order to uncover hints for the therapy of COVID-19, Shen and co-workers [20] created a framework for virus-drug association (VDA) identification using imbalanced bi-random walk, and Laplacian regularized least squares. Their proposed method performed reasonably well in terms of prediction. Also, their model in comparison with six state-of-the-art prediction models demonstrates superior prediction performance.

This paper deals with the last group, machine learning drug repurposing to predict new unknown associations among viruses and approved drugs. These prediction methods come in a wide range, starting from optimization to simple classical machine learning methods, e.g., random forest [21], SVM [22], and toward current state-of-the-art deep learning methods [23–25]. Most of those methods try to mimic or expand the matrix factorization approach. that is, decomposing a given matrix into two or more latent matrices. The original matrix can be estimated by multiplying these latent matrices. We call those methods in this paper as “**M**atrix **F**actorization based **D**rug **R**epurposing methods” (MF-DR). We define MF-DR fromally in Sect. 2.3

During our investigation on the subject, we realized that the MF-DR does not entirely fulfill the aim of DTI prediction and suffers from some drawbacks. First, the drug-target matrix is extremely sparse, and in most cases, the percentage of the available associations is less than one percent [1]. For example, most of the values in a row of drugs are zero, and there are just a single or a few entries with values equal to one. So, those methods consider an almost zero vector a non-sense feature vector. This sparsity causes another issue of a tremendous increase in the computation overhead and time. The complexities increase exponentially, which makes the method inapplicable. More importantly, the labels already exist in the feature matrix. In other words, there is data leakage in the training or learning process [26].

On the other hand, zero values in the drug-target matrix can have two entirely different interpretations of I) no association between each zero-value drug-target pair; II) unknown association between each zero-value drug-target pair. The last issue with those methods is the problem with matrix factorization itself. Matrix factorization is a dogmatic method that needs the number of columns or features to remain constant. When a new feature (e.g., a target) comes to the scene, the generated prediction model becomes useless. It will be necessary to re-run the learning process to have a new model with further information. The matrix factorization method comes from the recommender systems’ literature. Recommender systems are primarily helpful for recommending non-important subjects. In other words, a mistake has no harm in those fields, e.g., movie recommendation or another book based on the history of the previously purchased books; now, these borrowed methods aim to suggest solutions in the sensitive area of bioinformatics and drug repurposing.

Regarding the above issues with the matrix factorization paradigm, and having a proper prediction process, we believe that prediction happens based on the features and their similarities. Let’s assume there are some features like similarities among drugs as well as similarities among the targets. Moreover, there exist drug-target pair associations. It is better and closer to the real-world situation for prediction to consider the former similarities as the feature space and the drug-target pair associations as the labels. Doing this relieves us from the issues MF-DR deals with. For example, the feature space is not sparse anymore. Thus, it is better to avoid matrix factorization methods in the process of DTI prediction and generally in bioinformatics. Or at least use those matrix factorization methods with more caution.

**Fig. 1:**
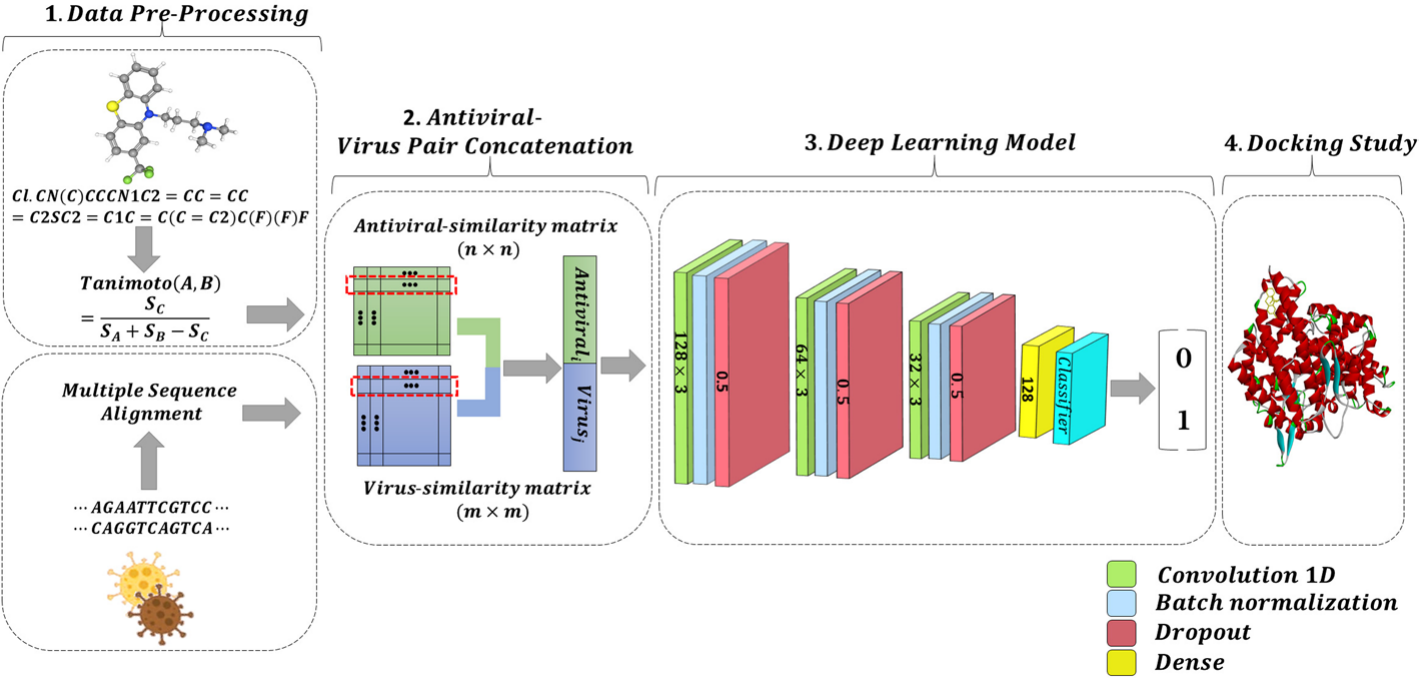
DRaW’s Framework. (1) Instead of applying to the virus-antiviral interactions, we use the model on the similarity data of antivirals and viruses. (2) Each sample of antivirals is concatenated with a virus. The results of the concatenation are the feature inputs to a deep network. (3) The deep model consists of four consecutive Conv1D layers with *Relu* activation function. Each of them is followed by batch normalization and dropout 0.5. Next, we use a dense layer after a flattened layer, followed by a dropout of 0.5. Finally, a dense layer with a sigmoid activation function acts as a binary classifier and predicts the interaction between the drug and protein. We compiled our model with Adam optimizer and binary cross entropy loss function. The prediction value is the association between the corresponding virus-antivirals. (4) Molecular docking study has been conducted on top-ranked drugs

We consider drug repurposing for COVID-19 as a state-of-the-art DTI research problem to proceed with the above analysis. We use three virus-antiviral interactions (VAIs) datasets. We call our proposal as **D**rug **R**epurposing-**a**nalytic **W**ay (DRaW). Figure 1 represents the DRaW framework. DRaW exclusively uses viruses’ and antivirals’ similarities as input features. In other words, in contrast with MF-DR methods, the sparse VAIs are not the input features of DRaW. It aims to predict VAIs. We compare our results with the published results of COVID-19 antiviral prediction [8, 18, 20, 27].

The results show DRaW outperforms the MF-DR methods. To be short, DRaW is fair and close enough to the prediction in the real world and laboratory investigations and has higher performance with less effort than the state-of-the-art methods. We have evaluated the top antiviral recommendations of DRaW for COVID-19 by docking study.

Moreover, to be sure of the results, we make an external validation on benchmark datasets [28] as well. The DRaW significantly outperforms the MF-DR. The evaluations prove the correctness of the predictions. Our top-ranking results are in harmony with the reported experimental studies on COVID-19. In contrast with previous suggestions on using matrix factorization (e.g., by [29] and [30]) MF-DR methods are not the best choice for drug repurposing studies.

## Materials and methods

### Datasets

**Table 1:** COVID-19 datasets statistics

Dataset	No of antivirals	No of viruses	Confirmed interactions
*DS*1	78	12	96
*DS*2	128	59	770
*DS*3	210	34	437

To show the benefit of direct use of similarity matrices, we have utilized three virus-antiviral datasets. The first dataset, *DS*1, was generated by [31] and contains 12 human RNA viruses and 78 antivirals, a total of 96 confirmed virus-antiviral associations. The second dataset, *DS*2, contains information on 59 viruses and 128 antivirals, with a total of 770 confirmed associations [20]. The third dataset, *DS*3, was gathered by [8] for COVID-19 treatments. The *DS*3 dataset comprises 34 human viruses such as RNA and DNA, HIV, and coronavirus. Also, it contains 210 specific and broad-spectrum antiviral drugs. There are 437 confirmed human drug-virus associations in this dataset. In addition, each of the above datasets has two corresponding similarity matrices, Virus similarity matrix (V) and Antiviral similarity matrix (AV). *DS*1 has V with size $12\times 12$ and AV with $78\times 78$, respectively. *DS*2 has V with size $59\times 59$ and AV with $128\times 128$, respectively. *DS*3 has V with size $34\times 34$ and AV with $210\times 210$, respectively. The similarity among viruses results from multiple alignments of genetic sequences with the “Multiple Alignment using Fast Fourier Transform” (MAFFT) algorithm [8]. To measure the similarity among antiviral pairs, the “Tanimoto coefficient” was used as the similarity metric [32]. Table 1 shows the statistics of the virus-antiviral datasets.

**Table 2:** Benchmark datasets statistics

Dataset	No of drugs	No of targets	Confirmed interactions
Enzyme	445	664	2926
GPCR	223	95	635
Ion channel	210	204	1476
Nuclear receptor	54	26	90

In addition to the virus-antiviral datasets, we have utilized benchmark datasets, as well. Benchmark datasets play an important role in comparing new techniques in the field of drug repurposing. The identification of drug-target interactions is a hot topic in drug discovery. Therefore, Yamanishi et al. [28] provided researchers in this area with “four classes of drug-target interaction networks in humans involving enzymes, ion channels, G-protein-coupled receptors (GPCRs) and nuclear receptors”. In addition, they made available drug structure similarity and target sequence similarity of the mentioned datasets. Table 2 presents the statistics of the benchmark dataset. Since then, these datasets have acted as external validation for the prediction of drug-target interactions.

### DRaW model

DRaW predicts the effective antiviral drugs for COVID-19 using the following objective function,

1 $$\begin{aligned} \min \sum _{i\in V_{test}}\sum _{j\in AV_{test}}\left[ I(i,j)-f\left( V(i,:),AV(j,:)\right) \right] . \end{aligned}$$

where *I* is the virus-antiviral association matrix, AV is the antiviral similarity, and V shows the virus similarity matrix. The indices *i* and *j* show *i*-th antiviral and *j*-th virus, respectively.

The typical matrix factorization methods decompose *I* into two latent feature matrices. In contrast with such scenarios, we do not decompose the *I* matrix. But we use the similarity matrices as the input features to the model. The model uses these similarity features to predict the VAIs. To do so, the model concatenates each row of AV with each row of V, and we update the above objective function as follows.

2 $$\begin{aligned} \min \sum _{i\in V_{test}}\sum _{j\in AV_{test}}\left[ I(i,j)-f\left( V(i,:)|| AV(j,:)\right) \right] \end{aligned}$$

which || shows the concatenation operation. Each row represents the concatenation of an antiviral similarity vector with a virus similarity vector. Thus, each row in the generated matrix shows a sample of antiviral-virus concatenation. We add the corresponding value of pair associations from *I* as the label of each sample. For example, the association of antiviral *i* and virus *j* is the (*i*, *j*)-th entry in the *I*. It is the label of the corresponding virus-antiviral pair. In short, each sample of virus-antiviral pairs is a combination of antiviral and virus similarity vectors, and its label is their corresponding VAI.

### MF-DR model

To show the higher performance of direct usage of similarity matrices as the feature space, we need to compare our results with conventional drug-target matrix factorization methods, which we call MF-DR here. To this end, we have used a technique in which virus-antiviral interactions are the input features of the samples in addition to similarity matrices. The goal of such methods is to decompose *I* into two latent factor matrices $U_{34\times f}$ and $W_{210\times f}$, where *f* is the number of the factors. The objective function is as follows.

3 $$\begin{aligned} \min \sum _{i\in V_{test}}\sum _{j\in AV_{test}}\left[ I(i,j)- \sum _{k=1}^{f}U_{ik}W_{jk} \right] \end{aligned}$$

or simply

4 $$\begin{aligned} \min _{U,W}\left\| I-UW \right\| _F^2. \end{aligned}$$

As is clear from the equations, the objective function 2 is different from the objective function 3. While the latter is matrix factorization, the former is a prediction using an input feature vector. Adding some regularization parameters to the objective function of matrix factorization methods is possible.

5 $$\begin{aligned} \min _{U,W}\left\| R-UW \right\| _F^2+\lambda _U\left\| U \right\| _F^2 +\lambda _W\left\| W \right\| _F^2+\mu (Similarity -terms) \end{aligned}$$

Tang et al. [8] proposed a type of MF-DR, and used similar objective functions to 5, to divide the drug-target pair matrix into two latent matrices. It is called IRNMF. Many of the methods mentioned in previous studies perform such objective functions. These methods are different in either handling the additional information, e.g., similarities or implementation algorithms (e.g., while [33] used an iterative optimization method, [25] used a deep model. Anyhow, both belong to the MF-DR).

### External method validation

In addition to executing the methods on the COVID-19 datasets, we evaluate the validity of our method in two ways. First, we apply DRaW and other methods to benchmark datasets [28]. Following that, we use the molecular docking approach on top-ranked antivirals suggested by DRaW to treat COVID-19. In the following subsections, we describe both external validations.

#### Evaluation of methods using benchmark datasets

We use four benchmark datasets of Enzyme, Ion Channel, GPCR, and Nuclear Receptor [28] to do the external validation of DRaW. The results of the benchmarks are from applying 5-fold cross-validation on benchmarks.

#### Molecular docking study

The anti-COVID-19 activity of each top-ranked drug predicted by DRaW in each dataset has been covered in a plethora of studies [34–36]. Nonetheless, for the validation of our proposed model’s prediction power, structure-based molecular docking experiments are carried out for some less-noticed drugs, such as triflupromazine hydrochloride, chlorpromazine, and loperamide. This technique is generally done as follows [37].

**Protein Preparation:** The crystal structure of the SARS-CoV-2 spike receptor-binding domain bound with ACE2 (PDB 6M0J) becomes the target protein for triflupromazine hydrochloride and chlorpromazine. Also, the crystal structures of SREBP1 (PDB 1AM9) are chosen as a target protein for loperamide and retrieved from the RCSB protein data bank database [38]. For the first complex (Spike-ACE2), both the spike protein and ACE2 were separated. Thus, chain A in the ACE2 structure is a target. Also, the SREBP1 dimer was separated. The procedure removes the HEATM and other solvent molecules from both structures using Discovery Studio. For energy minimization, we use the steepest descent method. In addition, we use the Swiss PDB Viewer (SPDBV) tool [39] to reduce the target proteins’ potential energy and obtain their most stable conformation. Then, we utilize the Autodock tools (ADT) to add polar hydrogen and assign Kollman charges to the energy-minimized target proteins. Afterward, the format of proteins is converted into PDBQT for molecular docking purposes.

**Ligand preparation:** The 3D-SDF structures of the top three ranked antiviral drugs were downloaded from the NCBI PubChem database [40] and were converted into the Protein Data Bank (PDB) format. Polar hydrogens and gasteiger charges were added to ligands. Also, root detection and choosing torsions from the torsion tree were done to rotate all the rotatable bonds. Ultimately, the PDB data of ligands was converted into PDBQT using the ADT 4.0 tool. We generate the Grid Parameter File (GPF) to locate “active site” residues. These residues actively participate in establishing stable interactions. SREs bind to the E-box site of SREBP1 using Glu332, His328, Tyr335 and Arg336 amino acids, which are highly conserved among helix-loop-helix proteins, as mentioned in [41, 42]. Thus, these amino acids were chosen as the most participant residues for docking the SREBP1-loperamide complex. Also, to determine the important residues in the bonding position of ACE2, the SARS-CoV-2 spike-ACE2 complex (PDB 6M0J) was visualized using the LIGPLOT+ tool [44]. The obtained pattern indicates that Asp30, Lys353, Gln24, Tyr83, Tyr41, Gln42, and Asp38 are the most important residues involved in forming this complex’s hydrogen bonds. For each docking job, we adjust the grid box in such a manner to enclose the active sites within it. For preparing the GPF of ACE2 protein, the grid box values are x-center=$-$37.26, y-center=32.197, z-center= $-$3.339, and x-points=34, y-points=98, and z-points=40. Also, for SREBP1, the center grid box is defined with 58.168, 27.345, and 127.623 as X-, Y-, and Z-coordinates, respectively. The grid points were 46, 52, and 74 in X-, Y-, and Z-coordinates. The grid point spacing is set to 0.375 angstroms for both of them. Also, the Lamarckian Genetic Algorithm (LGA) is the search method for performing molecular docking studies. All remaining parameters were set to the default.

**Ligands docking into proteins:** We have used the Cygwin terminal to set up and run the docking process. To this end, we have used both autogrid and autodock computations and done ten independent docking iterations for each antiviral drug. Final docked conformations were clustered based on the conformational similarities and root-mean-square positional deviation (RMSD) with a tolerance of 1.0 Å[44].

**Post-docking investigations:** the best poses correspond to the lowest binding energy ($\Delta$G) and orientation of the ligand within the defined binding pocket. Then, we used Biovia Discovery Studio Visualizer 2020 [45] to visualize and analyze the docking results to identify the intermolecular interaction forces and residues.

### Complexity analysis

In each epoch, the algorithm calls a pair of a single antiviral and a single virus. The number of antivirals in the train and test sets are $n_{tr}$ and $n_{te}$, respectively, and $n = n_{tr} + n_{te}$. The same goes with the number of viruses — $m_{tr}$ for the training phase and $m_{te}$ for the test phase, where $m = m_{tr} + m_{te}$. We consider the number of epochs for training set equal to *e*. Then if we assume that the time of each epoch is equal to $T_{ep}$, the complexity of the training phase for each antiviral-virus pair is equal to $O(eT_{ep})$, and the whole training phase for all the pairs — $n_{tr}m_{tr}$ — is $O(eT_{ep}n_{tr}m_{tr})$.

### Performance evaluation metrics

We compute the recall (sensitivity), specificity, precision, and F1-score metrics based on the following equations.

6 $$\begin{aligned} Recall= & {} \frac{TP}{TP+FN} \end{aligned}$$

7 $$\begin{aligned} Specificity= & {} \frac{TN}{TN+FP} \end{aligned}$$

8 $$\begin{aligned} Precision= & {} \frac{TP}{TP+FP} \end{aligned}$$

9 $$\begin{aligned} F1-score= & {} 2\times \frac{ precision\times recall }{precision+recall} \end{aligned}$$

Moreover, we used AUC-ROC, and AUPR. The former is a summary of the Receiver Operator Characteristic (ROC) curve which computes several pairs of sensitivity and $1-$ specificity by defining thresholds. The area under the curve (AUC) reports the capability of discrimination between the classes [46]. AUC-ROC is not proper for imbalanced datasets. Thus, we plot the Precision-Recall (PR) curve. It does not consider the true negatives (TN) samples and thus it is a common measure to report the classifier’s performance on the imbalanced data. We report the area under the PR (AUPR).

### Implementation

Figure 1 shows the DRaW’s framework. As mentioned in the figure’s description, it is a convolutional neural network. We use Adam as the optimizer with a learning rate equal to 0.001, $\beta _1=0.9$, $\beta _2= 0.999$, and $\epsilon = 10^{-7}$. The dropout rate is set to 0.5. The batch size is chosen by the number of samples per dataset. This hyperparameter for *DS*1 is equal to 8, and those for *DS*2 and *DS*3 are set to 32.

In order to minimize the error of the model for drug repurposing, we trained the model 10 times in 5-fold cross-validation and saved the recommended drugs in each fold based on the probability they obtained. Then, we choose the top recommended drugs with the best average rank.

## Results and discussion

This section reports the evaluation of our proposal. We utilized Tensor flow 2 and Scikit-learn [47] to do this. We compare DRaW with objective function 2 versus those methods which relied on matrix factorization. Figure 1 shows the scenario we have implemented. The methods using either the objective function 3, or 5 are IRNMF [8], GRNMF [33], IMC [48]. Thus, we give some statistics on the COVID-19 dataset. Moreover, we apply DRaW and IRNMF methods and a deep learning method (AutoDTI++ [27]) on the benchmark datasets [28]. The final part of the computational results deals with the top-ranked antivirals DRaW suggests for COVID-19.

### Performance analysis on COVID-19 datasets

**Table 3:** Comparison of DRaW with the other methods on COVID-19 datasets

Datasets	Methods	Recall	Specificity	Precision	F1 score	AUC-ROC	AUPR
DS1	IRNMF	0.750	0.614	0.182	0.2927	0.706	0.2927
	VDA-KLMF	**0.892**	0.544	0.300	0.367	**0.939**	**0.763**
	VDA-RWLRLS	0.562	**0.838**	0.141	0.225	0.885	–
	DRaW	0.642	0.836	**0.651**	**0.620**	0.822	0.589
DS2	IRNMF	0.801	0.728	0.220	0.345	0.816	0.2933
	VDA-KLMF	**0.826**	0.531	0.208	0.283	0.857	0.377
	VDA-RWLRLS	0.513	**0.826**	0.007	0.123	0.835	-
	DRaW	0.513	0.778	**0.441**	**0.463**	**0.865**	**0.458**
DS3	IRNMF	**0.741**	0.771	0.174	0.2820	0.809	0.222
	VDA-KLMF	0.863	0.522	0.163	0.233	0.866	0.391
	VDA-RWLRLS	0.519	0.843	0.067	0.118	0.862	–
	DRaW	0.538	**0.847**	**0.576**	**0.550**	**0.887**	**0.558**

This section provides the performance comparison of DRaW with MF-DR approaches on the COVID-19 datasets *DS*1, *DS*2, and *DS*3, introduced in Table 1. The methods are IRNMF [8], VDA-KLMF [18], and VDA-RWLRLS [20]. The IRNMF is a matrix factorization method, which as its authors reported outperforms other matrix factorization methods, i.e., GRNMF [33], IMC [48], CMF [49], and RLSMDA [50]. IRNMF returns the best result among these matrix factorization methods. It uses the similarity matrices and the main virus-antiviral matrix as the input to the procedure. VDA-KLMF, and VDA-RWLRLS belong to MF-DR and have shown high performance in COVID-19 drug repurposing. Thus, we chose these methods to report the performance of our proposal, DRaW. Table 3 reports the results. Performance evaluation metrics with the highest value have been highlighted in bold for each dataset DS1, DS2, and DS3. IRNMF and VDA-RWLRLS have low performance in comparison with the other two methods, VDA-KLMF and DRaW. For example, note their precision. As the results show, while VDA-KLMF has the highest AUC-ROC and AUPR for the smallest dataset (*DS*1), DRaW has the highest AUPR and AUC-ROC for *DS*2 and *DS*3. In addition, DRaW has the highest precision and F1 score in all datasets. Thus, DRaW presents the best results compared to all other matrix factorization methods. The results confirm that the MF-DR has lower performance than the non-MF-DR methods. As the results show, with an uncomplicated architecture,^1^ we reach a higher amount of performance and prediction compared to the state-of-the-art matrix factorization methods.

### Identifying potential drugs for COVID-19

**Table 4:** Recommended drugs for COVID-19 by DRaW on *DS*1

Rank	Drug
1	Remdesivir
2	Mycophenolic acid
3	Herbacetin
4	Chloroquine
5	Protein phosphatase 1
6	Ribavirin
7	Glycyrrhizin
8	Rhoifolin
9	Pentoxifylline
10	Phenothiazine

**Table 5:** Recommended drugs for COVID-19 by DRaW on *DS*2

Rank	Drug
1	Tamoxifen
2	Dalbavancin
3	Chlorpromazine
4	Clomipramine
5	Oritavancin
6	Toremifene
7	Telavancin
8	Teicoplanin
9	Amodiaquine
10	Chloroquine

**Table 6:** Recommended drugs for COVID-19 by DRaW on *DS*3

Rank	Drug
1	Triflupromazine Hydrochloride
2	Chlorpromazine
3	Loperamide
4	Thiothixene
5	Fluspirilene
6	Promethazine Hydrochloride
7	Ribavirin
8	Chlorphenozamine
9	Dasatinib
10	Clomipramine Hydrochloride
11	Fluphenazine
12	Astemizole
13	Imatinib
14	Terconazole

We extract DRaW’s top antiviral recommendations for each dataset. Tables 4, 5, and 6 show the top-ranked drugs suggested by DRaW for *DS*1, *DS*2, and *DS*3, respectively. According to data extracted from DrugBank, among the top 34 candidate drugs predicted by DRaW in three datasets, 13 drugs either have been or are under clinical trials for COVID-19, i.e., *remdesivir*, *chloroquine*, *ribavirin*, and *pentoxifylline* from *DS*1, *tamoxifen*, *chlorpromazine*, *toremifene*, *teicoplanin*, *amodiaquine*, and *chloroquine* from *DS*2, and *chlorpromazine*, *ribavirin*, and *Imatinib* from *DS*3.

**Fig. 2:**
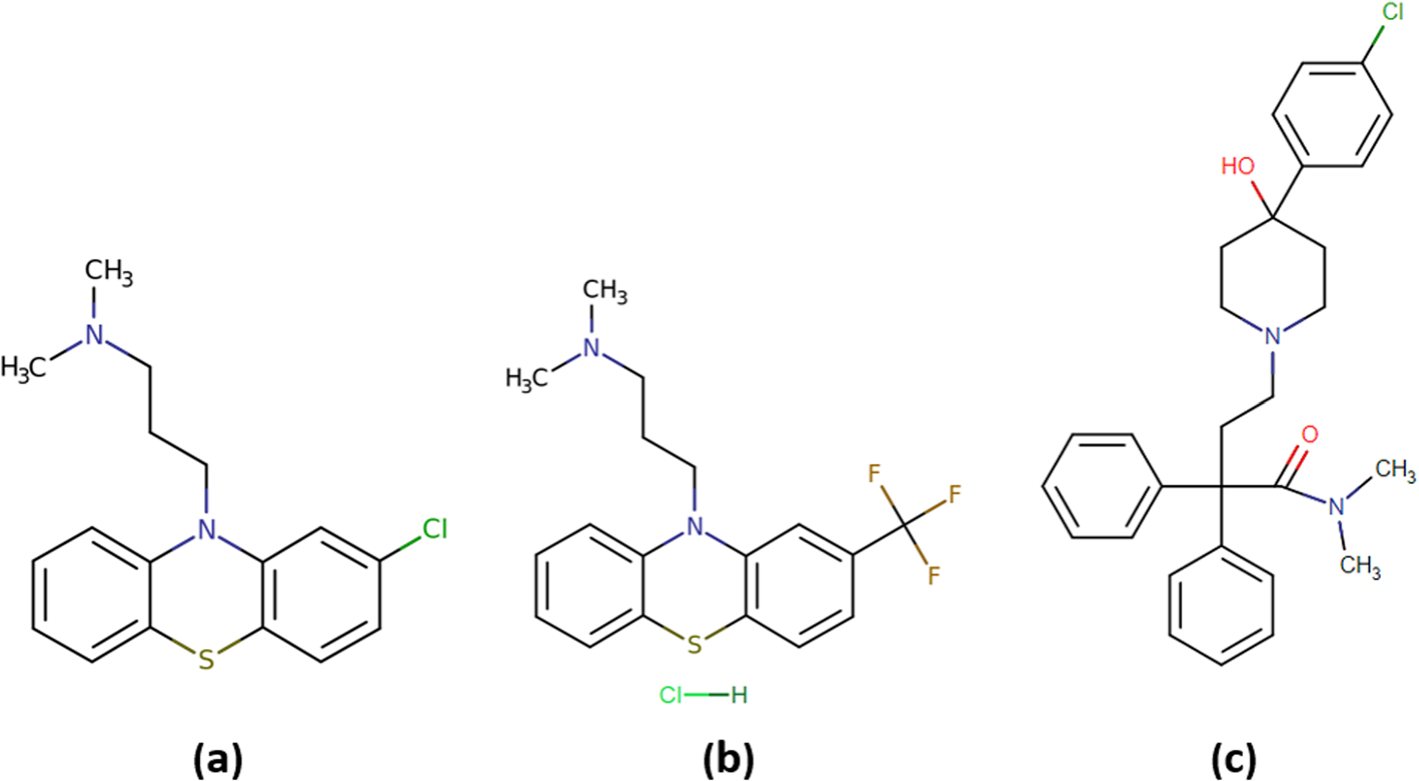
Two-dimension structure of top three-ranked drugs; **a** Chlorpromazine **b** Triflupromazine hydrochloride **c** Loperamide

In the first dataset, the top three predicted antiviral drugs are *remdesivir*, *mycophenolic acid*, and *herbacetin*. The top three predicted antiviral drugs in the second dataset are *tamoxifen*, *dalbavancin*, and *chlorpromazine*. Consequently, the top three antiviral drugs predicted in the third dataset are *triflupromazine hydrochloride*, *chlorpromazine*, and *loperamide*. We concentrate on examining the mechanisms of action (MOA) of *triflupromazine hydrochloride*, *chlorpromazine*, and *loperamide*. Because these drugs have had lower attention in the COVID-19 drug studies literature. Triflupromazine hydrochloride and chlorpromazine are neurotransmitter inhibitors in the typical antipsychotic class [51, 52]. The chemical structure and general properties of chlorpromazine are similar to those of triflupromazine hydrochloride, shown in Fig. 2a and b. These drugs have also shown antiviral and antimicrobial activity against several viruses and bacteria [53, 54]. Also, recent studies demonstrate that antipsychotic drugs can decrease the unfavorable evolution of COVID-19 infection, and consequently, repurposing antipsychotic drugs to treat COVID-19 has received a lot of attention [55–58]. The possible mechanism of these drugs against SARS-CoV-2 is to prevent virus entry into the host cells. Following spike-protein (S) binding to the angiotensin-converting enzyme 2 (ACE2), SARS-CoV-2 gains entry into the cell via the mechanism of clathrin-mediated endocytosis. Clathrin-mediated endocytosis is a process by which cargo-containing vesicles of SARS-CoV-2, which are coated by clathrin, pass from the cell membrane and are taken up into the cell [59, 60]. Chlorpromazine prevents clathrin migration from the cell surface, significantly inhibiting SARS-CoV-2 entry into cells  [61]. The same MOA happens for triflupromazine hydrochloride. In addition to the activities mentioned above, the current experimental *in-vitro* investigations have studied the affinity of some antipsychotic drugs to the ACE2 protein. The studies show the ability of these drugs to prevent the virus surface-anchored spike protein-mediated coronavirus entry. Their results state this class of drugs can significantly block SARS-CoV-2 binding to ACE2. Thus, antipsychotic drugs can inhibit the coronavirus entry into cells [62]. Loperamide, shown in Fig. 2c, is another of the top predicted antiviral drugs against coronavirus in our proposed model. Loperamide is an antidiarrheal drug that controls diarrhea symptoms by slowing gut motility [63]. Furthermore, this drug increases the activity of SREBF transcription factors which is one of the key regulators of lipid metabolizing enzymes [64]. The correlation between MERS-CoV replication and host cell lipid metabolism has been implicated. Therefore, manipulating cellular lipid metabolism to affect virus replication may be an appealing and notable approach to treating coronavirus infections [41]. The regulation of cellular lipid homeostasis and the synthesis of cholesterol and fatty acids are controlled by sterol regulatory element-binding proteins (SREBPs). In addition, multiple proteolytic processes have been reported for SREBP. The binding of SREB(s) to the specific sterol regulatory elements (SREs) in the cholesterogenic and lipogenic genes leads to the reversal of the virus-induced lipid hyper-biosynthesis [41, 65].

### Results on benchmark datasets

**Fig. 3:**
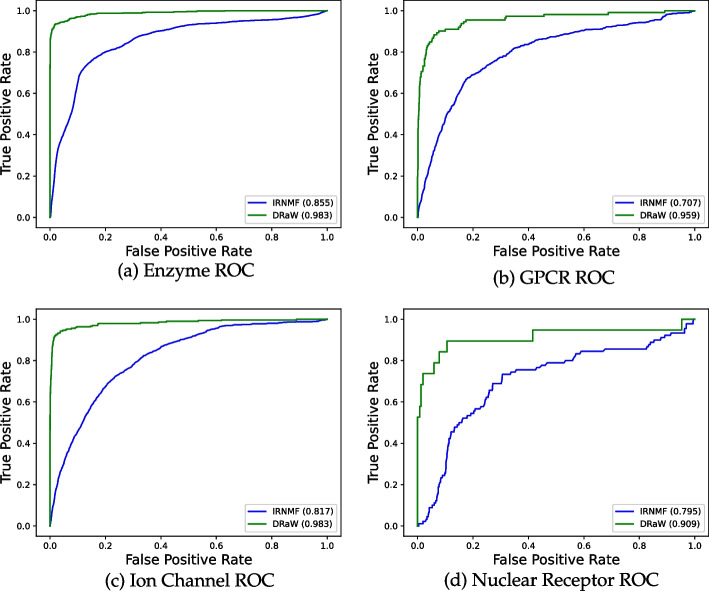
ROC of DRaW (green) and IRNMF (blue) on benchmark datasets

**Fig. 4:**
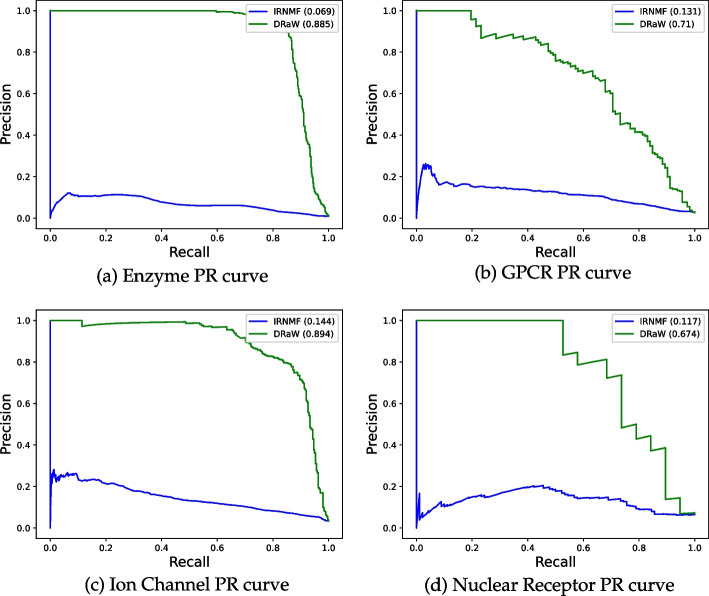
PR curve of DRaW (green) and IRNMF (blue) on benchmark datasets

**Table 7:** Comparison of DRaW with IRNMF [8], AutoDTI++ [27], and DNILMF [66] on benchmark datasets [28]

Dataset	Approach	AUC-ROC	AUPR
Enzyme	IRNMF	0.855	0.069
	AutoDTI++ ($S_{p}$)	0.90	0.82
	AutoDTI++ ($S_{d}$)	0.50	0.33
	AutoDTI++ ($S_{t}$)	0.84	0.77
	DNILMF	0.981	0.727
	DRaW	**0.983**	**0.875**
Ion Channel	IRNMF	0.817	0.144
	AutoDTI++ ($S_{p}$)	0.91	**0.90**
	AutoDTI++ ($S_{d}$)	0.49	0.50
	AutoDTI++ ($S_{t}$)	0.86	0.86
	DNILMF	0.982	0.831
	DRaW	**0.983**	0.886
GPCR	IRNMF	0.707	0.131
	AutoDTI++ ($S_{p}$)	0.86	**0.85**
	AutoDTI++ ($S_{d}$)	0.47	0.47
	AutoDTI++ ($S_{t}$)	0.85	0.83
	DNILMF	0.954	0.648
	DRaW	**0.955**	0.704
Nuclear Receptor	IRNMF	0.795	0.117
	AutoDTI++ ($S_{p}$)	0.87	0.84
	AutoDTI++ ($S_{d}$)	0.60	0.62
	AutoDTI++ ($S_{t}$)	0.87	0.84
	DNILMF	0.919	0.626
	DRaW	**0.954**	**0.883**

To be sure of the validity of our comparison, we applied DRaW, among other methods, on benchmark datasets, i.e., Enzyme, Ion Channel, GPCR, Nuclear Receptor [28]. We compare the DRaW with the IRNMF, AutoDTI++ [27], and DLILMF [66] methods. We already mentioned that IRNMF is an MF-DR method. Additionally, VDA-KLMF [18] is another MF-DR method. The authors borrowed the idea of dual-network integrated logistic matrix factorization (DNILMF) [66]. Thus, we ran the DNILMF to cover both mentioned methods. We chose AutoDTI++ due to it is a deep model. The authors considered the DTI matrix as the input to the model. Then they multiplied it by the feature vectors of drugs. Then, the computed matrix of this multiplication was fed to an autoencoder-based model. The autoencoder is a deep method. From the output of the model, they predicted the new DTIs. While they have used a deep method, their model suffers from considering DTIs as the input to the model. We mentioned this as a type of data leakage (and the main problem of matrix factorization methods) that makes the results unreliable. Nevertheless, we consider their results to compare. Table 7 shows the results. For each dataset, the highest AUC-ROC and AUPR values have been highlighted in bold. As the results show, our method outperforms the IRNMF on all datasets. The external validation shows our proposal’s power, which uses feature vectors rather than matrix factorization. The table needs more verification. The AUC-ROC metric shows that even an uncomplicated deep network on the similarity features outperforms the matrix factorization methods. However, if not all, most medical datasets are sparse matrices with a few ones and a massive number of trivial or zero values. More interestingly, although IRNMF may have a high value for the AUC-ROC, e.g., 0.855 for the Enzyme dataset (still lower than DRaW with an AUC-ROC higher than 0.98), its AUPR is tremendously negligible. This result shows that IRNMF predicts most of the values, if not all, as zero. This conversion to zero causes a fake high AUC-ROC and a low real AUPR. Thus, IRNMF and most matrix factorization methods cannot predict the correct ones. On the other side, considering similarity matrices as the feature space, as we have proposed in DRaW, leads to a higher and more acceptable AUPR. By comparing the DRaW with the AutoDTI++ versions, the former achieves a higher AUC-ROC on all datasets. However, DRaW has a higher AUPR in just two of the benchmark datasets and a lower in the other two. It is worth mentioning that these results of AutoDTI++ are polluted with data leakage. Lastly, DRaW has a higher AUC-ROC in all cases and a higher AUPR in Enzyme and Nuclear Receptor datasets. Anyhow, DRaW generally reaches a higher performance. In addition, diagrams in Fig. 3 present ROC curves, and diagrams in Fig. 4 present the PR curves of DRaW and IRNMF for benchmark datasets.

### Docking results

**Fig. 5:**
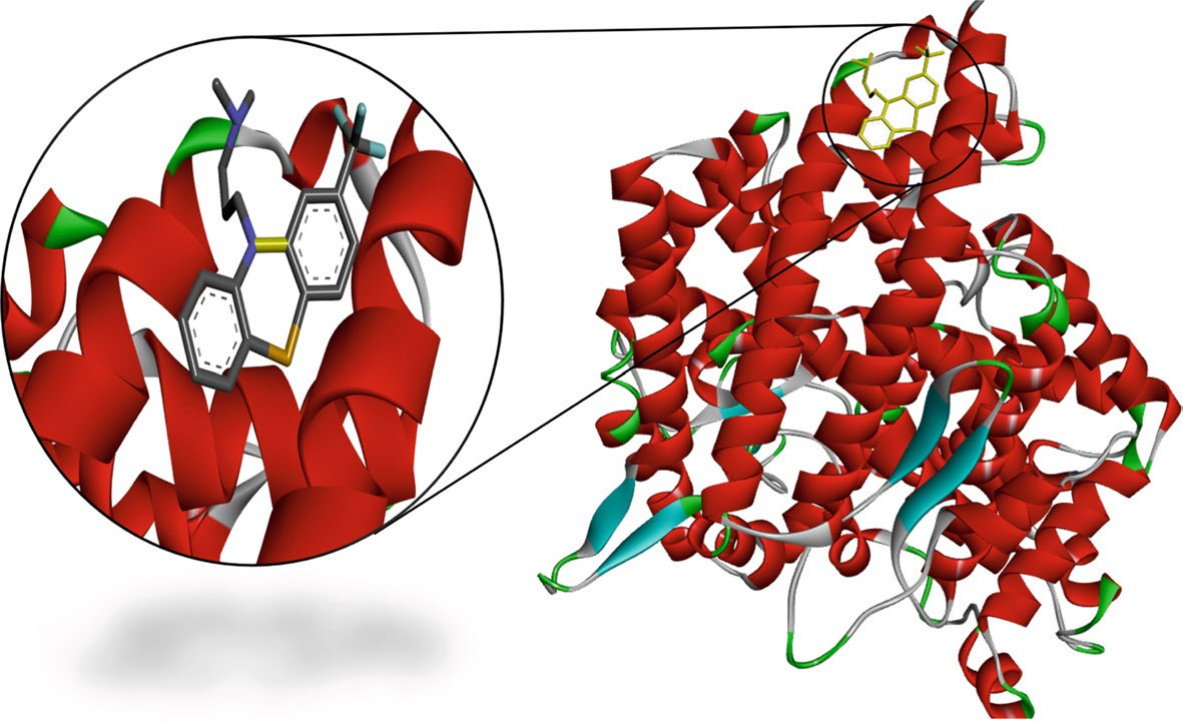
3D structure of the binding interaction between Triflupromazine hydrochloride-ACE2

**Table 8:** Docking results of top three ranked drugs recommended by DRaW against ACE2 and SREBP1

Compound Name	PUBCHEM CID	Molecular Formula	Docking Score (kcal/mol)	
			Ace2	SREBp1
Triflupromazine Hydrochloride	66069	$C_18 H_20 CIF_3 N_2 S$	− 7.0	–
Chlorpromazine	2726	$C_17 H_19 CIN_2 S$	− 6.1	–
Loperamide	3955	$C_29 H_33 CIN_2 O_2$	–	− 5.1

Table 8 shows the docking results of the three selected antivirals with the ACE2 and SREBP1.

**Fig. 6:**
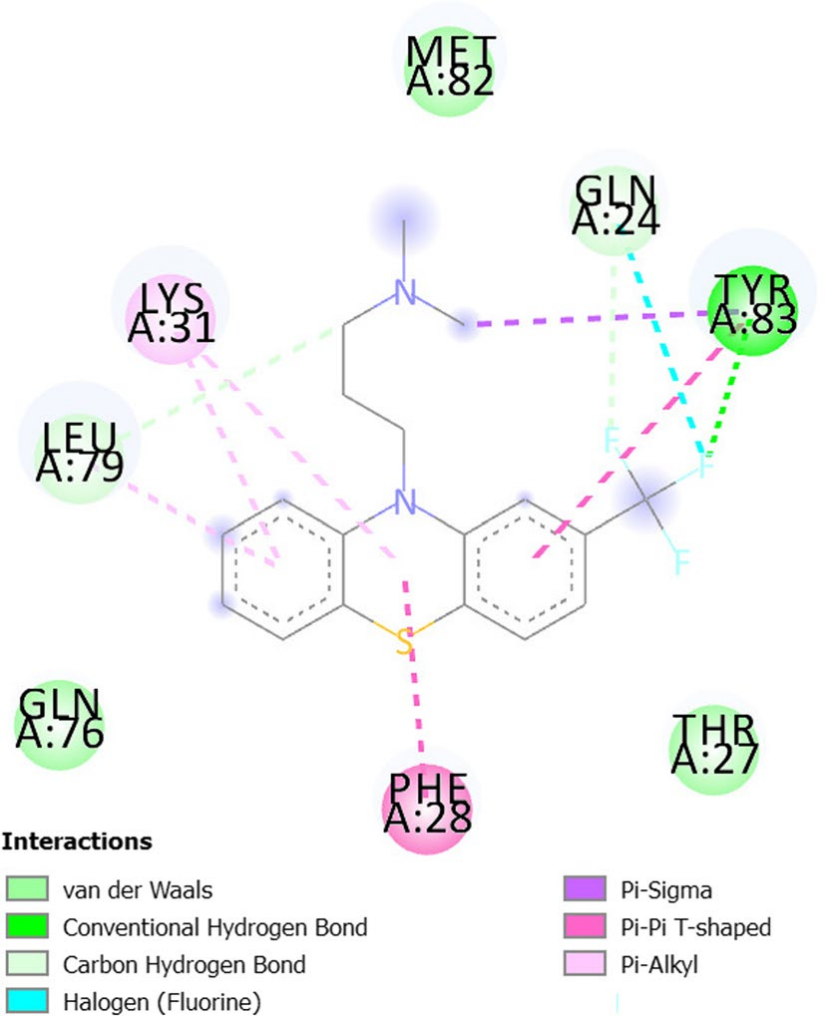
2D diagram for the residues incorporated in Triflupromazine hydrochloride-ACE2 interaction

**Fig. 7:**
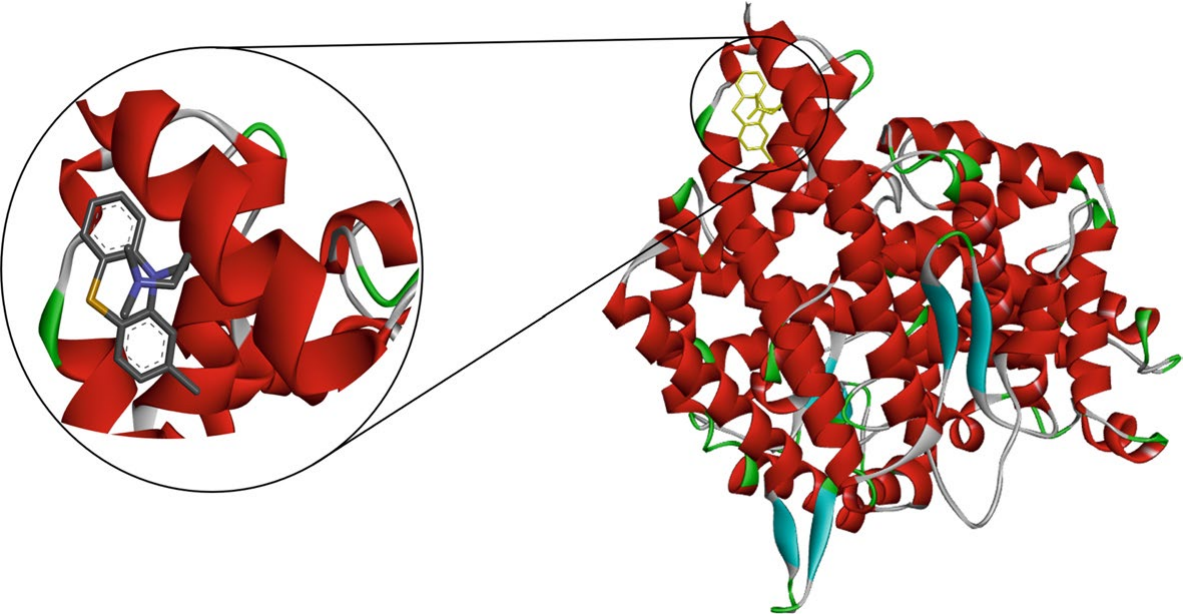
3D structure of the binding interaction between Chlorpromazine-ACE2

**Fig. 8:**
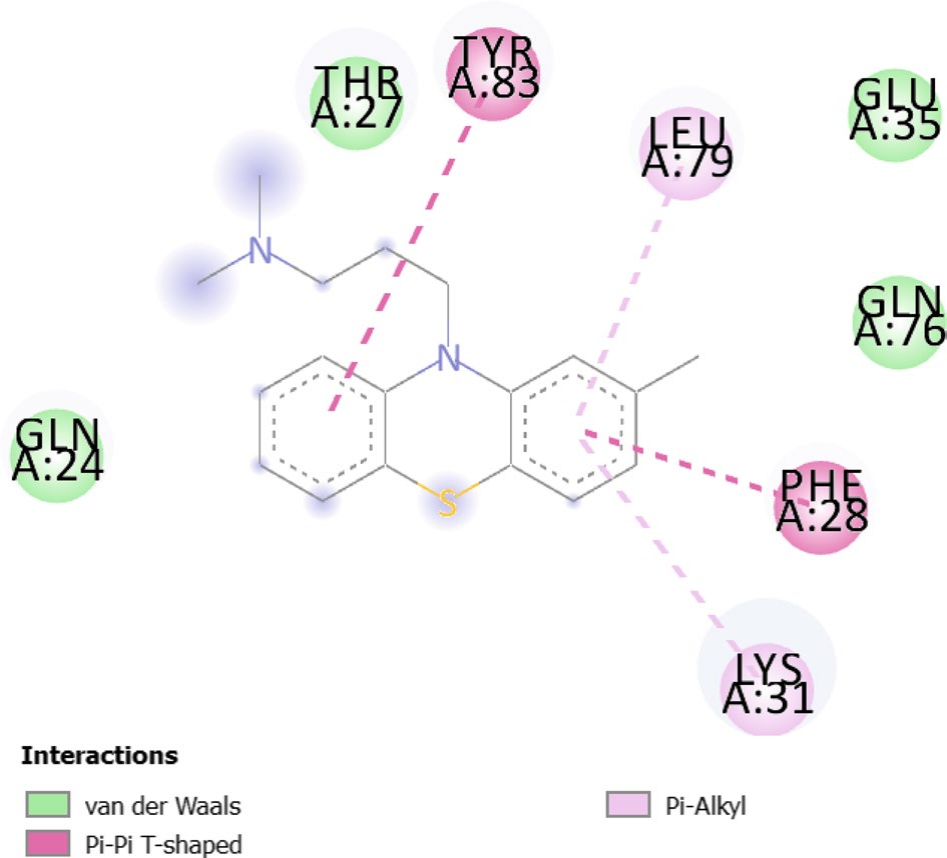
2D diagram for the residues incorporated in Chlorpromazine-ACE2 interaction

**Fig. 9:**
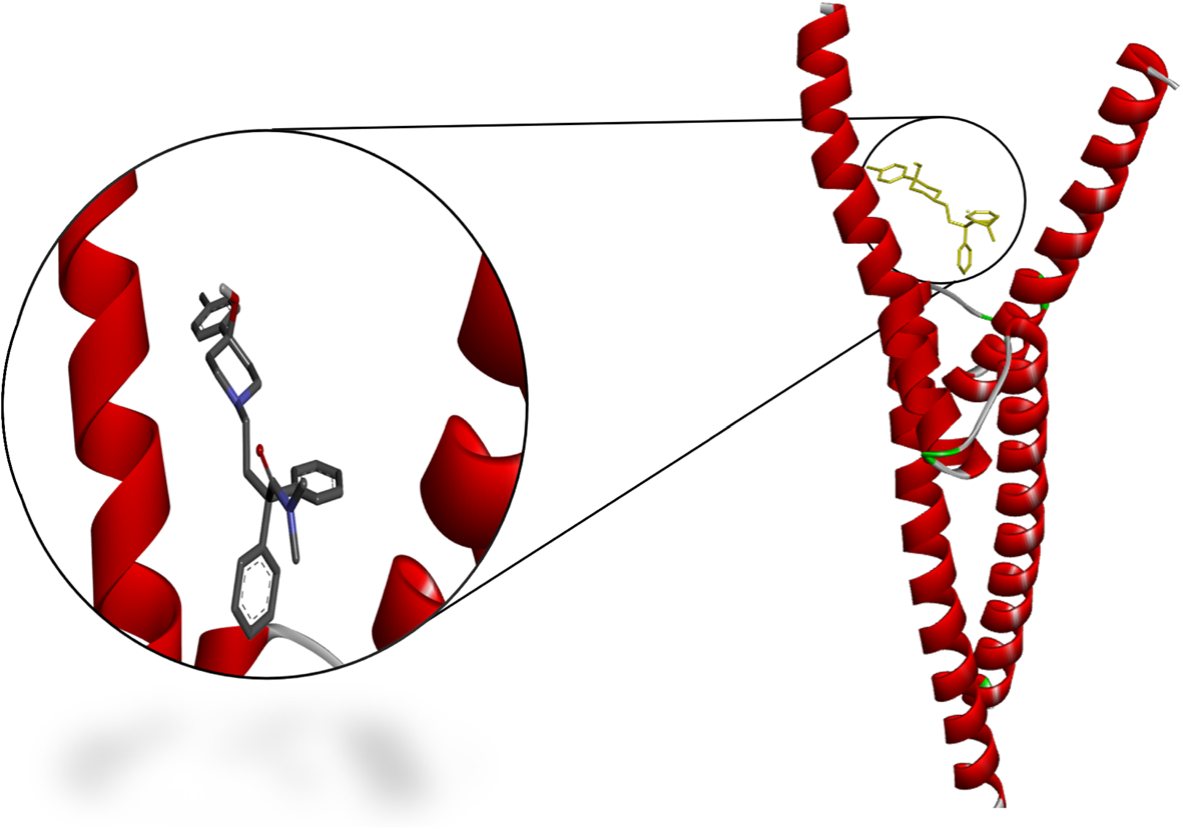
3D structure of the binding interaction between loperamide-SREBP1

**Fig. 10:**
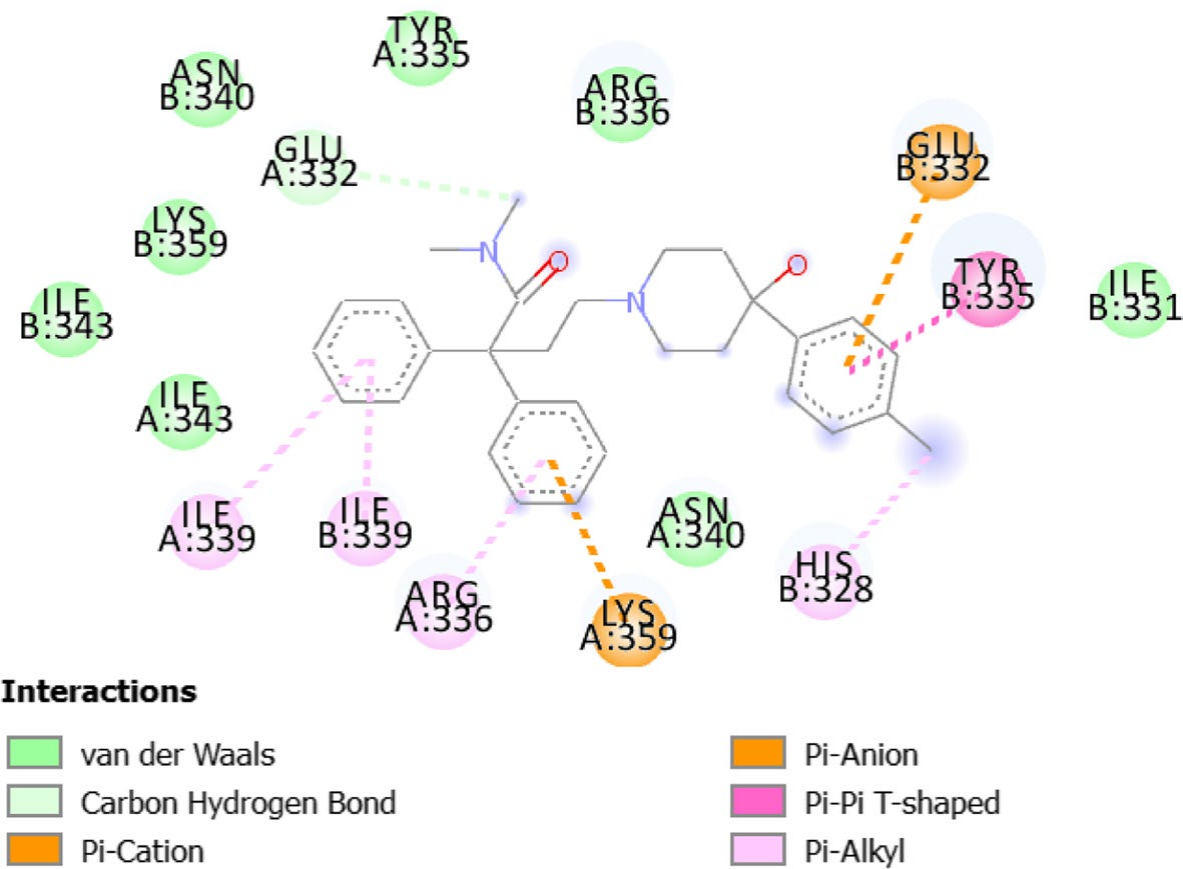
2D diagram for the residues incorporated in loperamide-SREBP1 interaction

All three drugs bind to their proteins with acceptable binding affinities and in the correct position. Triflupromazine hydrochloride binds to ACE2 by forming hydrogen bonds with Tyr83, and other interactions with Lys31, Leu79, Gln76, Phe28, Thr27, Gln24, and Met82, Figs. 5 and 6 show its 3D and 2D representations, respectively. As Figs. 7 and  8 show, the chlorpromazine binds to ACE2 by an intermediate of some van der waals interactions with Gln24, Thr27, Leu79, Glu35, Gln76, Lys31, and $\pi$-$\pi$ interactions with Tyr83, and Phe28. According to docking results, triflupromazine hydrochloride and chlorpromazine occupied the binding sites necessary for SARS-CoV-2; this explains the viral entry inhibition by these two drugs. Furthermore, as shown in Figs. 9 and 10, loperamide binds to the V-shape DNA-binding domain of SREBP1 by forming van der waals, $\pi$-$\pi$ and carbon-hydrogen bonds with *Ile*343, *Lys*359, *Glu*332, *Asn*340, *Tyr*335, *Arg*336, and *Ile*339. Therefore, loperamide can inhibit the DNA-binding domain activity of SREBP1 by physically blocking the SRE recognition site.

## Conclusion

In this paper, we deal with an analytical way of computational drug repurposing using machine and deep learning methods. Due to the tremendous time and cost of drug discovery, drug repurposing is an essential and undeniable part of this industry. Thus, many efforts of bioinformatic academic centers and research studies have concentrated on this subject. An important branch of drug repurposing utilizes matrix factorization methods borrowed from recommender systems. In this work, we analyzed the issues related to using such methods in drug repurposing studies. In addition, we have proposed a technique whose input feature consists of similarities and preliminary information on drugs or targets. In other words, we avoid sparse representations of drug-target interactions as the input vector. Our experiments on the COVID-19 dataset and external validation show that our proposal outperforms the matrix factorization methods.

## Data Availability

The data and code of DRaW are freely available at github.com/BioinformaticsIASBS/DRaW.

## Abbreviations

AUCROC: Area Under the Receiver Operating Characteristic curve
AUPR: Area Under Precision-Recall curve
AV: Antiviral similarity Matrix
DR: Drug Repurposing
DRaW: Drug Repurposing-analytic Way
DTI: Drug-Target Interaction
GPCR: G Protein-Coupled Receptors
I: Virus-Antiviral Interaction matrix
MF: Matrix Factorization
MF-DR: Matrix Factorization-Drug Repurposing Methods
ROC: Receiver Operating Characteristic

## Footnotes

1 We do not use the complicated deep learning architecture in this paper. Our aim in this paper is to show the problems with matrix factorization.
